# Using a Stages Model to Reveal the Politics in the Health Policy Process: Comment on "Modelling the Health Policy Process: One Size Fits All or Horses for Courses?"

**DOI:** 10.34172/ijhpm.2023.8066

**Published:** 2023-06-20

**Authors:** Rod Sheaff

**Affiliations:** Peninsula School of Medicine and Dentistry, University of Plymouth, ITTC Plymouth Science Park, Plymouth, UK

**Keywords:** Policy Process, Policy Cycle, Policy Formation, Rationality, Politics of Health, Stages Model

## Abstract

Models of the health policy process have largely developed in isolation from political studies more widely. Of the models which Powell and Mannion’s editorial considers, a stages model of the policy process offers a framework for combining these specifically health-focused models with empirical findings and more general explanatory models of the policy process drawn from other political studies. This commentary uses a stages model to assemble a bricolage which combines some of these components. That identifies a further research task and suggests ways of revealing in more life-like ways the politics involved in the health policy process: that is, how that process channels wider, often conflicting, non-health interests, actors, policies, conflicts, ideologies and sources of power from outside the health system into health policy formation, and introduces non-rationality.

 Powell and Mannion finish their editorial^1^ by asking whether we should analyse health policy processes in the same way as other policy processes (a ‘One Size Fits All’ approach) or use more health-specific explanatory models: ‘Horses for Courses.’ This commentary answers, ‘Both, of course.’ The question is, how to combine them, which leads as we shall see to a further question: how to reveal the politics within the health policy process.

 The editorial shows how the health policy process has largely been analysed in isolation from political studies more widely. Only a few studies apply a ‘model’ or ‘theory’ of the health policy process, and the editorial itemises seven such models. Correctly but not very informatively, the cited studies altogether list ten sources of health policy —actors, institutions, problems, networks and so on. As the editorial says, listing them is not the same as explaining the connections among these factors, nor their connections with the resulting health policies. In passing the editorial notes that Jones et al^2^ used ‘bricolage’ (patch-work assembly) to combine models, bringing in actors, ‘content,’ processes and more. This comment builds upon the editorial by taking that idea further.

 As an empirical (not normative) model of the policy process, a stages model offers a framework for combining the models and sources which Powell and Mannion list with findings and explanations from political studies more widely about what factors drive policy processes. Stages models (of which there are variants) typically identify such main stages in policy formation as: problematisation; the problem’s entry into the policy agenda; policy adoption; implementation; and evaluation.

 During problematisation some aspect of (in this case) health or health-care becomes salient to political actors (organisations, parties, the media) as a ‘problem’ requiring a policy response. At one extreme catastrophes such as pandemics, invasion or natural disasters force policy-makers to respond. More typically, effects from slower, longer-running trends, including those which social determinants of health models itemise, gradually accumulate to the point where their consequences become a salient practical problem for some political actor or coalition. These slower trends show more clearly that problematisation is something agential, undertaken by interest groups, lobbies, political parties, and the media among others. Often such trends (eg, prison overcrowding) are a problem for some actors but for others a matter of indifference or even a political or economic opportunity. If the trend seems to present an opportunity, political actors or coalitions of them discover, select, exaggerate or invent ‘problems’ to justify policies which they want to pursue for extrinsic electoral, ideological or economic reasons. Opposing actors employ different discourses to frame the emerging problem in terms that legitimate their own preferred ‘solution.’^3,5^ The advocacy coalition (ACF), institutional analysis and development and narrative policy frameworks emphasise the agential, normative and discursive character of issue problematisation, the Areas of Conflict framework, ACF and multiple implementation theory its often conflictual character.

 As to whether a problem and its supposed solution enter the policy-making agenda, Lukes’ classic analysis^5^ contrasts three scenarios. Policy makers might be unable or unwilling to acknowledge the problem at all, either because they dislike the policy responses which it seems to demand (climate change denial might be an example) or because the discourses through which they articulate policy problems cannot accommodate it (eg, cannot accept that female illiteracy is objectionable). Alternatively policy makers may acknowledge the problem but refuse to let decision-making institutions consider it, pre-empting the adoption of any new policy at all.^6^ Third, they might acknowledge the problem and add it to their decision-making agendas with a view to deciding what policy ‘solution’ to adopt. Again all three responses often have political, ideological and economic motives originating outside the world of health or healthcare.

 Entering the policy agenda may lead to policy adoption: policy-makers decide what policy to adopt, including what outcomes they expect the policy to produce (or remove), and through what mechanisms and resources (financial, legislative, human, informational or others). Their attempts to win the support of conflicting interest groups, including sceptical voters, often lead policy-makers to adopt policy which is ambiguous in order to appeal to conflicting groups simultaneously,^7^ or which substitutes rhetorical, spurious or vague goals^8^ to mask the more contested goals that policy-makers actually wanted to achieve. To satisfy different interest groups the policy may include disparate, even incompatible, and redundant elements (eg, accommodating private medicine alongside a national health system) or exclude elements likely to arouse opposition, including practically efficacious elements (eg, sex education for school-children). It may rest upon on false assumptions about what caused the problem (eg, victim-blaming assumptions about poverty) or be chosen more to symbolise that policy-makers are addressing the problem rather than for any practical impact.^9^ Again, it may be chosen to promote wider ideological goals such as neo-liberalism or ‘third way’ social democracy. Policies which contain such elements thus do result from attempting to recruit support from multiple political actors (as ACF, institutional analysis and development, and group theory models say), but actors with partly-conflicting interests (as the Areas of Conflict, ACF, multiple implementation theory, and social movement models note).

 Powell and Mannion note that models of the policy process often stop short of considering implementation. Extensive but again largely separate research shows, among other things, that ‘street level’ managers often re-interpret or deflect a policy when implementing it,^10^ at times for reasons extrinsic to the rationale for the policy itself. They often implement policy selectively. They add workarounds for unworkable or (in their view) counter-productive parts of the policy, including parts that conflict with other concurrent policies. To that extent, the policy that is implemented differs from what policy-makers adopted, with correspondingly different outcomes. Furthermore, the contexts which produce implementation deficits typically vary between places, organisations, professions and care groups.

 When it feeds back into the problematisation, agenda-entry and policy-adoption stages, evaluation helps (as punctuated equilibrium theory suggests) drive the policy cycle, whether by legitimating or discrediting policies pursued for other reasons (‘policy-based evidence’) or by diagnosing scientifically the causes or absence of policy failure or implementation failure.

 Such a bricolage implies, firstly, that some models of the policy process contradict each other less than first appears. Whether the sources of health policy lie in shared values or value-conflicts depends partly on whether the one focuses on the health policy community as the source of health policies, or also upon outside actors with their extrinsic ideological or economic motivations. Nevertheless, another line of development that Powell and Mannion’s editorial suggests would be to examine systematically what contradictions between the models nevertheless remain and, since contradictions would prevent a simple bricolage of those models, what evidence would decide between them. The above bricolage also suggests why health policy usually, but not always, only changes incrementally (as punctuated equilibrium theory suggests), and why the ‘policy windows’ that MSF describes only open occasionally: because policy adoption requires unpredictable successive stages of the policy cycle to align. It also suggests why the policy process often produces unintended consequences and ‘bad’ policy, and why the criteria of badness are in part relative to one’s political standpoint, economic and social interests. (A structural adjustment programme may be good from the World Bank standpoint and bad from that of a Zimbabwean tenant farmer).

 Realist evaluation has become an established method for diagnosing policy and implementation success or failure. Another direction for developing Powell and Mannion’s editorial would be to consider how the realist idea of a context-mechanism-outcome configuration relates to the models reviewed. One link is the concept of ‘programme theory’: the assumptions, which any policy implicitly embodies, that certain mechanisms (institutions, incentives, information flows etc) will produce the outcomes that the policy-makers wanted, provided perhaps that certain contextual conditions exist.^11^ Powell and Mannion’s editorial, and the above bricolage suggested by it, indicate why a policy’s programme theory is often slippery to define. For the health policy process often produces policies which are ambiguous, internally inconsistent, exclude or conceal controversial policy mechanisms, make false causal assumptions, are discursively blind to parts of the original policy problem and its causes, and at times intended more to symbolise than launch a practical attempt to deal with the problem. And that is when the underlying policy aims (motives, intended outcomes) are expressed at all.

 Powell and Mannion’s editorial used Weible and Sabatier’s selection of models of the policy process.^12^ It might be argued, though, that the selected models are themselves somewhat isolated from the political theories which consider not only how established policy processes work but also how the policy processes themselves originated and what shapes them in turn. Whilst giving very different answers, the ‘grand’ political theories — pluralism, elite theory, Marxism, structuralism, institutionalism, social contract theory and so on — have tended to explain the origins and working of policy processes in terms the acquisition, maintenance and uses of political power: who exercises it, who is subjected to it, how it is maintained and exercised when interests conflict, and to whose benefit. To paraphrase Hobbes^13^ only slightly, maintaining and exercising political power generally involves an admixture of force, fraud and finance to further some actors’ interests and frustrate others. Policy processes are therefore to be explained also in those terms, hence by factors which include actors and interests largely outside the health system.

 In this light, Powell and Mannion’s editorial also reveals that many models of the healthcare policy process are doubly apolitical. A focus on rationality and shared values is perhaps more understandable in the health policy than, say, the foreign policy process since the aim of maintaining population health is less contested and scientific evidence is increasingly brought to bear upon it. However models of the healthcare policy process tend to be isolated, too, from the studies which reveal the contested, interest-driven character of much the health policy process itself (names such as Klein^14^ and Navarro^15^ spring to mind), considering just the actors within the health system. Also the models often underplay the extent to which policy advocates and policy-makers inject wider ideologies (eg, the Washington consensus, populism, socialism with Chinese characteristics and so on), and political and socio-economic interests that transcend the health sector, into the health policy process; and therefore underplay the partly non-rational, even anti-rational, character of policy processes. Such factors strongly influence which actors the health policy process involves and excludes, why them and not others, what processes are used (and not used) to decide policy, and which prospective policy contents are taken seriously or ignored.

 To explain characteristics which the health policy process shares with policy processes in other domains thus requires a high-generality model which explains the nature of policy processes themselves in terms of political power, economic interests and geopolitics. At this high level of generality ‘one size fits all’ policy processes including health. That general model then has to be qualified (not replaced) with additional explanations of how and why policy processes in a particular polity (say China, the United States, the European Union or a low-income country) are distinct special cases of that general model; and then how and why the health policy process is a still more special case within that: a ‘horses for courses’ approach. But whatever the level of generality, the health policy process depends on more than actors, ‘contents’ and processes within the health system alone. To understand the health policy process it is necessary to reveal who uses it, for what, and under what constraints. That is, how it channels wider, often conflicting, non-health interests, actors, policies, conflicts, ideologies, and sources of power from outside the health system into health policy formation, and introduces non-rationality. One must see the politics in it.

## Ethical issues

 Not applicable.

## Competing interests

 Author declares that he has no competing interests.
